# Efficacy of Selpercatinib in Non-small Cell Lung Cancer With Bilateral Internal Auditory Canal Metastases: A Case Report

**DOI:** 10.7759/cureus.81182

**Published:** 2025-03-25

**Authors:** Diana Moreira-Sousa, Manuel Morgado, Maria S. Valente

**Affiliations:** 1 Department of Pulmonology, Local Health Unit of Cova da Beira, Covilhã, PRT; 2 Faculty of Health Sciences, University of Beira Interior, Covihã, PRT; 3 Pharmaceutical Services, Local Health Unit of Cova da Beira, Covilhã, PRT; 4 4 RISE-Health, Department of Medical Sciences, Faculty of Health Sciences, University of Beira Interior, Covilhã, PRT; 5 Faculty of Health Sciences, University of Beira Interior, Covilhã, PRT

**Keywords:** brain neoplasms, cerebral metastasis, neoplasm metastasis, non-small-cell lung cancer, oncogenic mutations, ret fusion, selpercatinib

## Abstract

Selpercatinib is a selective rearranged during transfection (RET) inhibitor approved for treating RET fusion-positive non-small cell lung cancer (NSCLC), demonstrating high efficacy in central nervous system involvement. This case report describes a 65-year-old woman with stage IV lung adenocarcinoma who, after progression on third-line therapy, developed severe neurological symptoms, including hypoacusis, headache, and dizziness, attributed to cerebral and bilateral internal auditory canal metastasis. This study received a favorable opinion from the ethics committee of the Cova da Beira Local Health Unit and informed consent was obtained from the patient in question. Next-generation sequencing identified a RET fusion mutation, leading to the initiation of selpercatinib as a fourth-line treatment. The patient exhibited significant clinical improvement within one week of therapy, including complete hearing recovery. Adverse effects were limited to elevated hepatic transaminases and QT interval prolongation, both of which were effectively managed through dose adjustments. The response to selpercatinib was sustained for over 31 months, at which point new brain metastasis developed, which was possible to address with whole-brain radiotherapy while maintaining targeted therapy with selpercatinib. This case highlights a rare presentation of bilateral auditory canal metastasis in NSCLC with RET fusion, following the failure of platinum-based chemotherapy and immunotherapy. The prolonged progression-free survival and favorable tolerability of selpercatinib, after dose modifications, underscore its potential as an effective treatment option for patients with central nervous system metastasis.

## Introduction

The therapeutic options for non-small cell lung cancer (NSCLC) have experienced significant advancements in recent years, with an increasing focus on targeted therapies based on genetic and molecular alterations, particularly in cases of locally advanced and metastatic disease [1-3].

The rearranged during transfection (RET) proto-oncogene encodes a receptor tyrosine kinase involved in embryonic development [4]. RET fusion mutations result in ligand-independent activation and abnormal expression of RET, promoting uncontrolled cellular proliferation and oncogenesis [4]. These mutations are identified in approximately 1%-2% of non-squamous NSCLC cases and are strongly associated with brain metastases, with a reported lifetime prevalence of up to 46% in affected patients [5-7].

Selpercatinib, a highly selective RET inhibitor, is approved for the treatment of locally advanced or metastatic RET fusion-positive NSCLC in patients who have not previously been treated with a RET-targeted therapy [8]. A Phase III clinical trial that included patients with advanced NSCLC demonstrated a significant improvement in progression-free survival (PFS) and a considerable intracranial disease response in individuals treated with selpercatinib compared to those receiving chemotherapy [9]. However, real-world data on long-term outcomes of selpercatinib in patients with RET fusion-positive NSCLC and symptomatic central nervous system (CNS) metastases remain limited.

We present a clinical case of a patient with stage IV NSCLC and symptomatic CNS metastases who achieved a striking and sustained symptomatic and radiological response following treatment with selpercatinib. This clinical case was previously presented as a conference abstract at the International Association for the Study of Lung Cancer (IASLC) 2022 World Conference on Lung Cancer in August 2022.

## Case presentation

**Figure 1:**
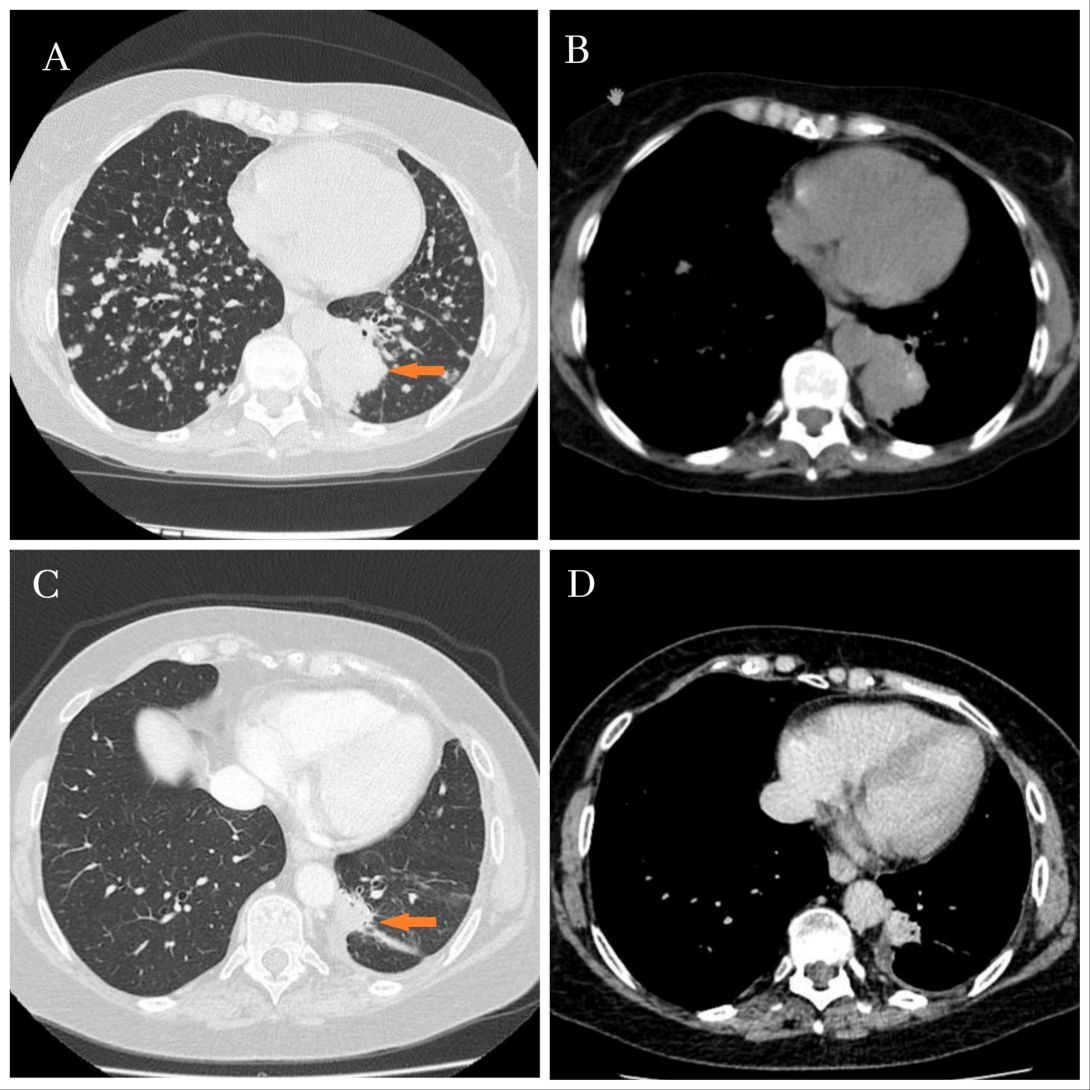
Thoracic computed tomography scan at diagnosis and after six cycles of the first-line therapy. Thoracic computed tomography scan at diagnosis, with a left paravertebral mass of 45 mm (arrow in A) and multiple millimetric nodules that were assumed as metastasis in both lungs (A and B). After six cycles of first-line therapy, a partial tumor response was observed, characterized by a significant reduction in the primary tumor size (arrow in C) and near-complete regression of bilateral micronodules (C and D).

A 65-year-old never-smoker woman with an Eastern Cooperative Oncology Group (ECOG) performance status of 0 [10] and a personal medical history of well-controlled hypertension, without other prior comorbidities, was referred for consultation due to complaints of fatigue and an unintentional weight loss of 3 kg. Diagnostic workup, including a thoracic computed tomography (CT) scan, revealed a heterogeneous 45 mm mass, a solitary 11 mm pre-carinal lymphadenopathy, ipsilateral pleural effusion, and multiple millimetric nodules consistent with bilateral metastases (Figures 1A, 1B). A subsequent biopsy confirmed stage IVa (cT4N2M1a, TNM 8th edition [11]) lung adenocarcinoma with acinar and micropapillary patterns. Extrathoracic metastatic sites were excluded. At diagnosis, molecular testing was negative for epidermal growth factor receptor (EGFR), anaplastic lymphoma kinase (ALK), and ROS1 alterations. The patient was initiated on first-line systemic therapy with a platinum-based doublet, completing six cycles with a partial tumor response according to Response Evaluation Criteria in Solid Tumors (RECIST) criteria, followed by maintenance therapy with pemetrexed, with sustained stability (Figures 1C, 1D) [12].

**Figure 2:**
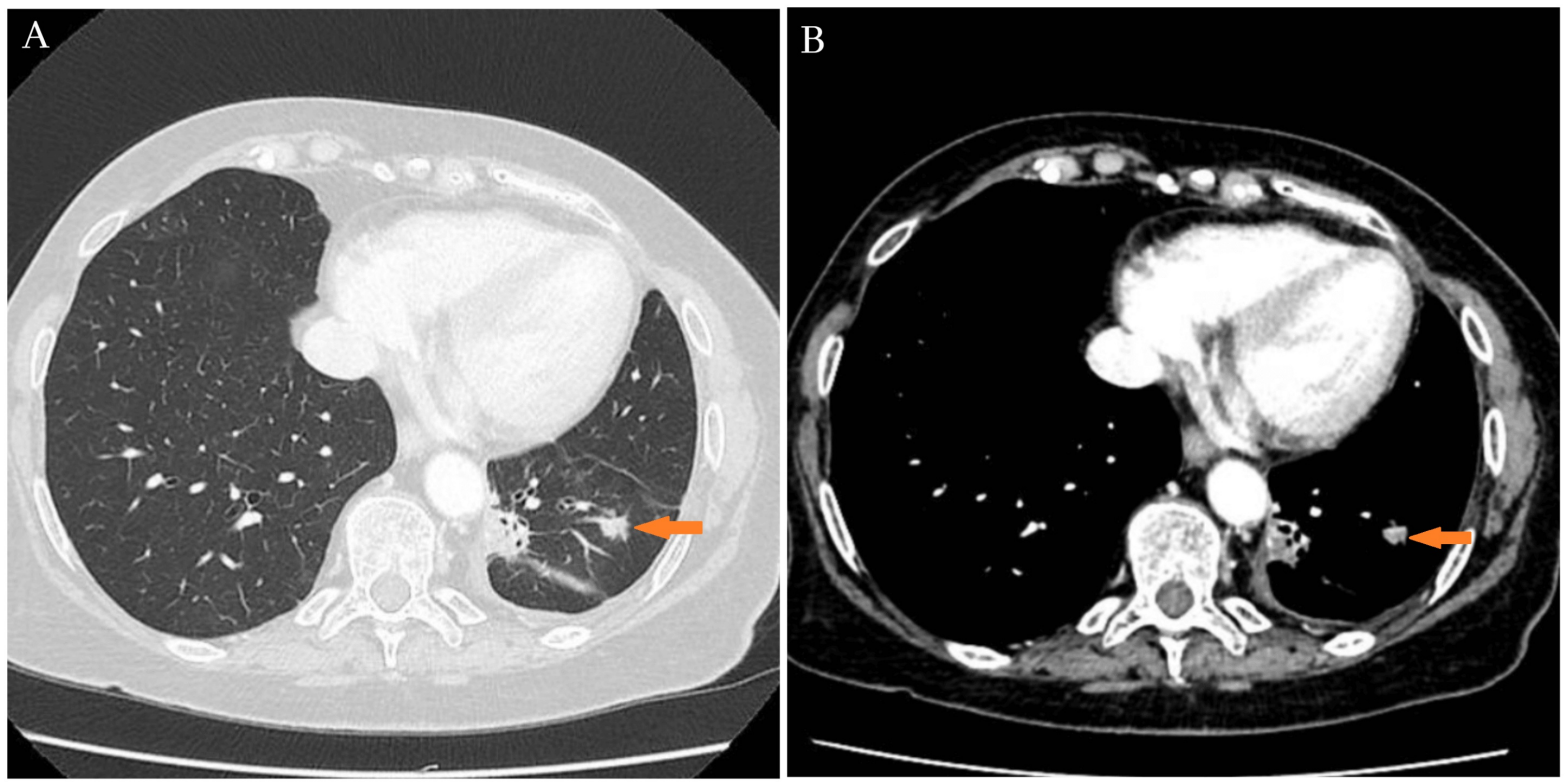
Disease progression at four-year follow-up. Disease progression in November 2020, with a new growing nodule (arrows) reaching 11 mm of the longest diameter, with a 21% increase in the sum of the longest diameter of target lesions.

Approximately four years later, in November 2020, disease progression was identified with the emergence of a new growing nodule measuring 11 mm in its longest diameter (Figures 2A, 2B).

A re-biopsy of the paravertebral nodule confirmed adenocarcinoma, with a programmed death-ligand 1 (PD-L1) expression of 5%. Next-generation sequencing (NGS) revealed the presence of a RET gene fusion. At this point, second-line off-label therapy with cabozantinib was initiated; however, it was discontinued after one month due to significant adverse effects, including severe asthenia, nausea, and thrombocytopenia. Subsequently, third-line therapy with pembrolizumab was initiated.

**Figure 3:**
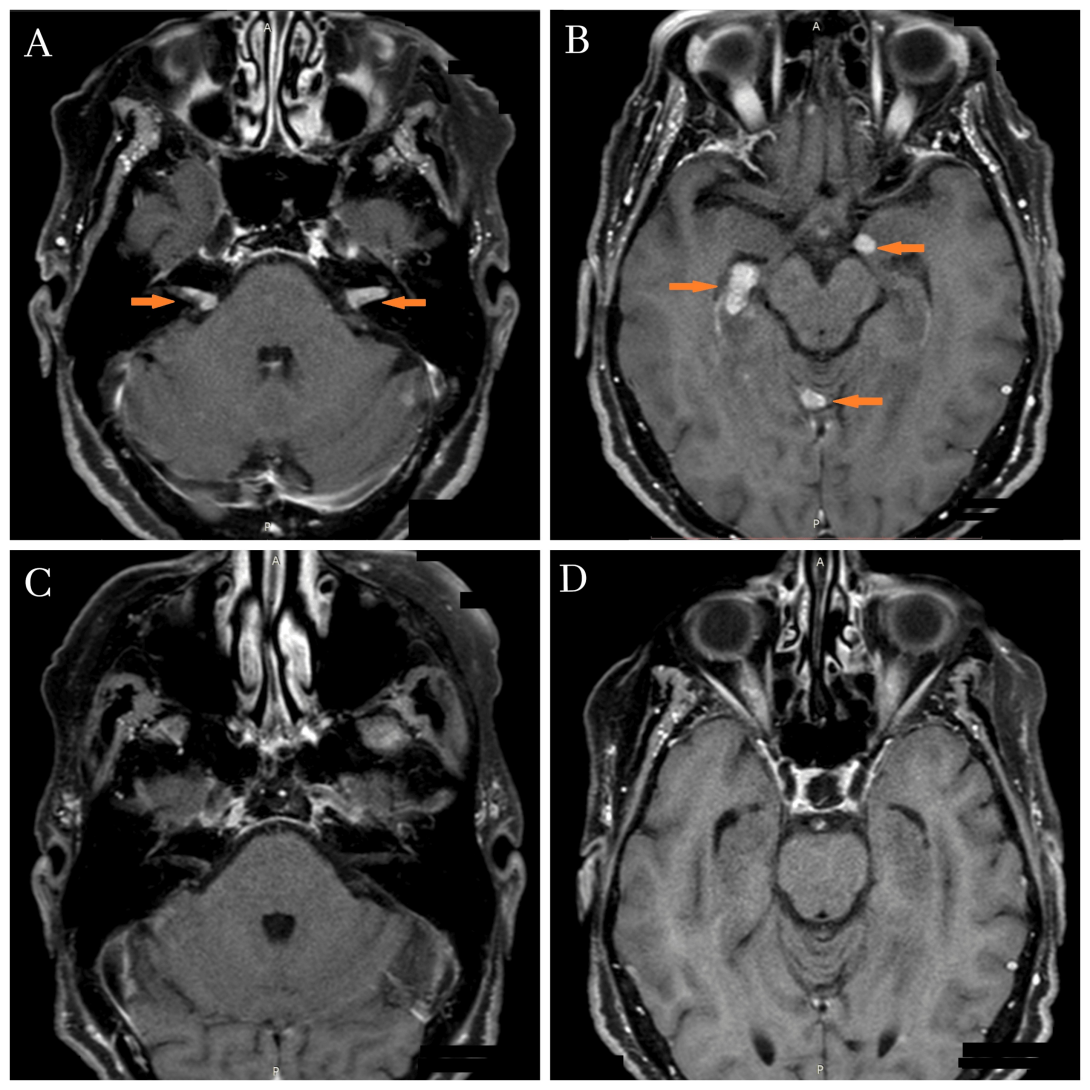
Cerebral magnetic resonance imaging (MRI) performed before and after the initiation of treatment with selpercatinib. Gadolinium-enhanced cerebral MRI revealed an enhancing lesion within the internal auditory canal (arrows) (A) and bilateral expansile lesions in the cerebellar hemispheres (arrows) (B), measuring less than 15 mm. At the three-month follow-up after initiating selpercatinib, MRI revealed near-complete resolution of the metastatic lesions, demonstrated by the minimal gadolinium enhancement in comparison to the baseline (C and D).

The disease remained stable for 10 months before the patient developed progressive neurological symptoms of bilateral hypoacusis, headache, and dizziness, without the presence of other complaints such as fever, photophobia, or nausea. Considering the preceding oncological context and the progressive and persistent nature of the reported symptoms, disease progression with CNS involvement was the primary suspicion. Vascular, inflammatory, and infectious diseases, as well as peripheral etiologies of vertigo, were considered less probable. Cranial magnetic resonance imaging (MRI) confirmed the suspicion, by revealing multiple expansive lesions in both cerebral and cerebellar hemispheres, as well as bilateral internal auditory canal (IAC) involvement, with gadolinium enhancement (Figures 3A, 3B), highly suggestive of tumor progression in this clinical context. Considering the therapeutic options available at the time and the patient's ECOG performance status of 1 [10], fourth-line treatment with selpercatinib (160 mg twice daily) was initiated in December 2021. Surprisingly, within just one week of treatment, the patient presented significant symptomatic improvement, including complete hearing recovery and a substantial reduction of dizziness and headache complaints. At the three-month thoracic assessment, radiological evaluation demonstrated a reduction in the previously detected pulmonary nodule and stability of the paravertebral lesion. Simultaneous follow-up cranial MRI revealed near-complete resolution of multiple supratentorial and infratentorial nodular lesions, with the absence of gadolinium enhancement, except for a faint residual signal at the cisternal segment of the right eighth cranial nerve (Figures 3C, 3D).

The disease remained stable until July 2024, following 31 months of continuous treatment with selpercatinib, when three new intracranial lesions were identified, accompanied by clinical manifestations of paresthesia and reduced muscular strength in the lower limbs. Considering the context of the sustained stability of the thoracic disease and the absence of metastases in other sites, the oncology group decided to proceed with local ablative therapy with hippocampal avoidance whole-brain radiotherapy (HA-WBRT).

Regarding adverse effects during treatment with selpercatinib, an initial asymptomatic liver enzyme elevation led to a dose adjustment (120 mg b.i.d.), and at five months, QTc prolongation (502 ms) required a temporary pause and resumption at 80 mg b.i.d. Additional adverse effects observed shortly after treatment initiation included peripheral edema and hypotension, which were managed by discontinuing antihypertensive medications and starting treatment with a loop diuretic. In subsequent years, the patient demonstrated good tolerance to the reduced selpercatinib dose.

At 37 months of treatment with selpercatinib, the patient maintains an ECOG performance status of 2 [10], with stable thoracic disease, while the outcomes of HA-WBR are under evaluation. The treatment timeline and symptom progression of the present case report are summarized in Table 1.

**Table 1:** Summary of treatment timeline and symptom progression. b.i.d., twice daily; CNS, central nervous system; ECOG, Eastern Cooperative Oncology Group; HA-WBRT, Hippocampal Avoidance Whole-Brain Radiotherapy

Timepoint	Treatment	Symptom progression and clinical response
Diagnosis (Stage IV)	Platinum-based doublet (six cycles) followed by maintenance Pemetrexed	Partial tumor response, stable disease
Progression (four years)	Cabozantinib (off-label)	Significant adverse effects, discontinued after one month
Third-line therapy	Pembrolizumab	Stable disease for 10 months
Further progression	Neurological symptoms, CNS metastases detected	Bilateral hypoacusis, headache, dizziness
Fourth-line therapy	Selpercatinib (160 mg b.i.d.)	Rapid improvement within one week, complete hearing recovery
Three-month follow-up	Selpercatinib	Near-complete resolution of CNS lesions
31-month follow-up	Selpercatinib + HA-WBRT	New brain metastases treated, thoracic disease remains stable
37-month follow-up	Selpercatinib (dose adjusted)	Stable thoracic disease, ECOG 2, ongoing evaluation

## Discussion

This case describes a patient with lung cancer progression, presenting cerebral, cerebellar, and bilateral internal auditory canal (IAC) metastasis at the third line of therapy, with a RET-fusion mutation identified after nodule re-biopsy. Although IAC and cerebellopontine angle metastasis from solid tumors are rare, lung cancer is the most common malignancy associated with bilateral IAC involvement [13]. Clinical manifestations indicative of IAC metastasis, particularly in patients with a prior oncological history, include acute-onset hypoacusis, tinnitus, vertigo, and facial palsy [13]. The prognosis of patients with bilateral IAC metastasis is usually poor, with a reported rate of death of 66.7% within six months [13]. However, in this case, a fast and sustained response to targeted therapy was observed, with neurological improvement - including hearing recovery - within one week and survival of 37 months after initiating selpercatinib. A previous work at the phase I/II trial of selpercatinib (LOXO-292) documented a similar rapid clinical response in a patient with a RET-fusion-positive NSCLC with leptomeningeal metastasis, further supporting our findings [14].

The Phase III clinical trial demonstrated high efficacy in stage IV patients with CNS metastasis, as selpercatinib - a potent RET-fusion inhibitor with brain penetration - showed a PFS benefit of 16.1 months [9]. In the presented case, the disease progression under Pembrolizumab occurred nearly after the European regulatory approval of selpercatinib as the preferred therapeutic option for patients with advanced/metastatic NSCLC positive for RET rearrangement mutations, after first-line chemotherapy [8,15]. Considering the multiplicity of the cerebral lesions and the possibility of systemic target treatment with selpercatinib, which exhibits high CNS penetration, the decision was made to postpone brain RT and initiate a systemic therapy trial. Remarkably, this case exceeded the outcome reported in the Phase III clinical trial, achieving a PFS of 31 months, when cerebral oligoprogression occurred, and was accessed with local treatment with HA-WBRT, while maintaining selpercatinib to extend the benefit of the current line of systemic therapy, according to guidelines [9,15,16]. Additionally, Phase I/II and Phase III trials excluded patients with evolving symptomatic neurological conditions [9,17]. However, in the presented case, despite the presence of progressive symptoms, a rapid and effective response was observed, further supporting the fast-acting efficacy of this RET inhibitor. Nevertheless, while this case presents compelling findings regarding the efficacy of selpercatinib in managing CNS and IAC symptomatic metastasis, it is essential to recognize that these results are based on a single clinical observation.

After 37 months of treatment with selpercatinib, it is important to emphasize that the patient continues to demonstrate good treatment tolerance, with a performance status of 2, and a good quality of life. Indeed, the patient was diagnosed with stage IV NSCLC nearly nine years ago, highlighting the real-life impact of recent advancements in oncological treatment options and care. In addition to selpercatinib being generally well tolerated, supportive and palliative care, along with careful monitoring of adverse effects, were considered essential components of the treatment. All the adverse effects observed were previously documented in the LIBRETTO-001 trial and described in the European Medicines Agency (EMA) product information [7,8] and did not implicate definitive treatment suspension. Dose adjustments with de-escalation according to EMA product information permitted treatment tolerability and continuation [8]. We also recorded an unusual decrease in arterial blood pressure after starting selpercatinib, as hypertension is one of the most common lateral effects [8].

## Conclusions

Selpercatinib is a highly effective treatment for patients with NSCLC RET-fusion-positive tumors, achieving significant results, particularly in cases involving the CNS. This case report documents the response of CNS metastasis with rare bilateral internal auditory canal involvement, illustrating the striking and sustained symptomatic and radiological response to the treatment, further highlighting the efficacy and safety of selpercatinib.
